# Development of an automated CBCT‐based simulation‐free platform for expedited palliative radiotherapy on a conventional linear accelerator

**DOI:** 10.1002/acm2.14612

**Published:** 2024-12-23

**Authors:** Riley C. Tegtmeier, Edward L. Clouser, Quan Chen, Courtney R. Buckey, Suzanne J. Chungbin, Christopher J. Kutyreff, Jose S. Aguilar, Amber L. Labbe, Brooke L. Horning, William G. Rule, Sujay A. Vora, Yi Rong

**Affiliations:** ^1^ Department of Radiation Oncology Mayo Clinic Arizona Phoenix Arizona USA; ^2^ Department of Radiation Oncology University of South Florida Health Morsani College of Medicine Tampa Florida USA; ^3^ Department of Radiation Oncology St. Jude Children's Research Hospital Memphis Tennessee USA

**Keywords:** CBCT, expedited radiotherapy, palliative radiotherapy, SFRT, simulation‐free radiotherapy

## Abstract

**Background:**

Conventional approaches for emergent or expedited palliative radiotherapy (RT) involve the application of cumbersome vendor‐provided solutions and/or multiple patient appointments to complete the RT workflow within a compressed timeframe.

**Purpose:**

This report delineates the clinical development of an in‐house, semi‐automated Cone‐beam computed tomography (CBCT)‐based simulation‐free platform for expedited palliative RT on conventional linacs, intended to supplant existing techniques employed at this institution.

**Methods:**

The internal software, termed SimFree Wizard (SFW), was engineered utilizing a C#‐based application programming interface integrated within the treatment planning system (TPS). Generated scripts were compiled as stand‐alone executables, with a graphical user interface (GUI) customized via an integrated development environment. The platform was conceived as a framework for accelerated CBCT‐based RT, bypassing the requirement for standard simulation imaging. SFW employs full automation where feasible to minimize user intervention, supplemented by graphical instructions for tasks requiring manual execution. During development, relevant temporal metrics from 10 end‐to‐end tests for palliative spine RT were quantified. User feedback was solicited via a simple questionnaire assessing the overall platform usability. Automated in‐house secondary verification software was developed for validation of the TPS‐calculated monitor units (MUs).

**Results:**

The mean duration for workflow execution was 41:42 ± 3:18 [mm:ss] (range ∼37–46 min). SFW satisfactorily generated simple, multi‐field CBCT‐based 3D treatment plans within seconds following delineation of the desired treatment area. User feedback indicated enhanced usability compared to previously employed solutions. Validation of the secondary verification software demonstrated accurate results for palliative spine RT and other simple cases wherein the dose calculation point resides in a predominantly homogenous medium.

**Conclusion:**

A novel in‐house solution for expedited CBCT‐based RT was successfully developed, facilitating completion of the entire workflow within approximately 1‐hour or less for simple palliative/emergent scenarios. Overall, this application is expected to improve the quality and safety of palliative RT while greatly reducing workflow duration, thereby improving access to palliative care.

## 1 INTRODUCTION

Constituting an estimated 30%–70% of all administered treatment regimens, radiotherapy (RT) with palliative intent plays an integral role in the symptomatic alleviation of skeletal and soft‐tissue metastases.^1^, ^2^, ^3^, ^4^ While palliation has numerous indications, it is primarily applied to address pain from debilitating complications of bony malignancies, including spinal cord compression, hypercalcemia, and pathologic fracture.^5^, ^6^ Notably, spinal metastases afflict 3%–5% of all cancer patients,^7^ with metastatic spinal cord compression incident in an estimated 15% of patients with advanced disease.^8^ For these conditions, palliative RT has proven effective in providing pain relief, preserving functionality, and maintaining skeletal integrity with minimal risk of serious adverse side effects.^9^, ^10^ Palliative RT is conventionally delivered in 1 to 10 fractions following standard treatment planning and delivery workflows, including physician consultation, acquisition of a simulation computed tomography (CT) scan, delineation and plan generation, plan check, and quality assurance, and so forth.^4^, ^11^ However, this approach is increasingly onerous, requiring multiple appointments and several days to complete while potentially introducing logistical obstacles for both the patient and RT department.^2^ In the palliative setting where symptom management is paramount, this delay is suboptimal, as prompt completion of RT is essential to lessen patient suffering and derive maximum treatment efficacy.^3^, ^12^ Moreover, a subset of these patients presents with acute medical conditions necessitating immediate, emergent RT (such oncological emergencies are defined by “conditions arising from a reversible threat to an organ function requiring radiation treatment within several hours of diagnosis”).^13^, ^14^, ^15^ In general, patient inconvenience and access issues resulting from conventional workflows may ultimately lead to referrers opting against palliative RT altogether.^3^, ^16^ To expedite the process and mitigate the burden of palliative care (and make emergent care feasible), a common approach is to bypass acquisition of a standard simulation CT scan and instead generate treatment plans based on additional imaging data as available. This method is referred to as simulation‐free RT (sim‐free RT).^4^

Historically, 2D planning utilizing fluoroscopic or radiographic acquisitions was prevalent for palliative RT, facilitating same‐day treatment while requiring minimal additional resources.^3^ With this approach, simple treatment strategies, such as parallel‐opposed field‐based planning guided by bony anatomy, are feasible, and palliative plans can be rapidly generated without a conventional simulation image.^2^, ^17^ However, these plans are generally derived from crude, manual calculations, whereas modern imaging technology provides the opportunity to leverage 3D anatomical data to enhance target delineation and dosimetric accuracy.^17^ Cone‐beam CT (CBCT)‐based sim‐free RT techniques have been explored for palliative care settings.^12^, ^17^, ^18^, ^19^ In the RT practice environment, the ability to acquire 3D datasets at the treatment unit has created significant efficiency potential for “scan, plan, and treat” clinical workflows based on the “anatomy‐of‐the‐day”.^12^, ^17^ Moreover, recent hardware and software advancements have significantly improved the attainable image quality (and dosimetric accuracy) of CBCT systems,^20^, ^21^ addressing a key limitation to clinical implementation in the past.^19^ Among the proposed approaches in the literature (including magnetic resonance imaging‐^22^, ^23^, ^24^ and diagnostic CT‐based^2^, ^3^, ^4^, ^11^ methods), the CBCT‐based sim‐free RT technique is perhaps the most readily applicable in clinical practice.

Nonetheless, obtaining suitable imaging data for planning is only one consideration when integrating sim‐free RT, as expediting palliative care presents unique patient safety and workflow challenges. Furthermore, in the emergent RT setting, access to standard resources is constrained as treatment predominantly occurs beyond normal clinical hours, leading to a heightened threat of treatment errors given the condensed timeline and complexity of care.^25^ This facility has historically employed a vendor‐provided unplanned treatment mode (UTM—Varian Medical Systems, Inc., Palo Alto, CA) available on the linac console to deliver emergent, port film‐based 2D plans for palliative RT after‐hours. However, this approach necessitates significant user intervention and is susceptible to an amplified risk of misadministration, given the intricacy of the process and infrequency of care. Thus, it is desirable to establish streamlined, sim‐free RT workflows to improve patient safety and alleviate the burden on staff and clinical resources. To this end, automation has been identified as an effective strategy to enhance efficiency and quality in RT,^26^ and access to vendor‐provided scripting interfaces has facilitated the development of automated workflows for a variety of RT use cases.^27^, ^28^, ^29^

The objective of this report is to detail the clinical development of an automated in‐house, CBCT‐based simulation‐free platform for the delivery of expedited palliative RT. This online solution leverages a commercial scripting application (Eclipse Scripting Application Programming Interface (ESAPI), Varian Medical Systems, Inc.) and previously developed in‐house software and databases (and their associated APIs) to integrate all imaging, planning, and delivery steps into a single treatment session with a duration of 1‐hour or less. While initially designed to replace unplanned treatment mode (UTM) and better assist on‐call teams in the safe administration of emergent RT after‐hours (primarily for the treatment of spinal metastases), this strategy can be extended to accelerate delivery of standard palliative RT as well. The practical development framework of this tool and initial observations from phantom‐based testing (for palliative spine RT) are described herein, accompanied by consideration for prospective applications of this software moving forward. Additionally, results from one of the initial clinical patient treatments performed via application of this platform are presented.

## 2 METHODS AND MATERIALS

### 2.1 Software overview

**FIGURE 1:**
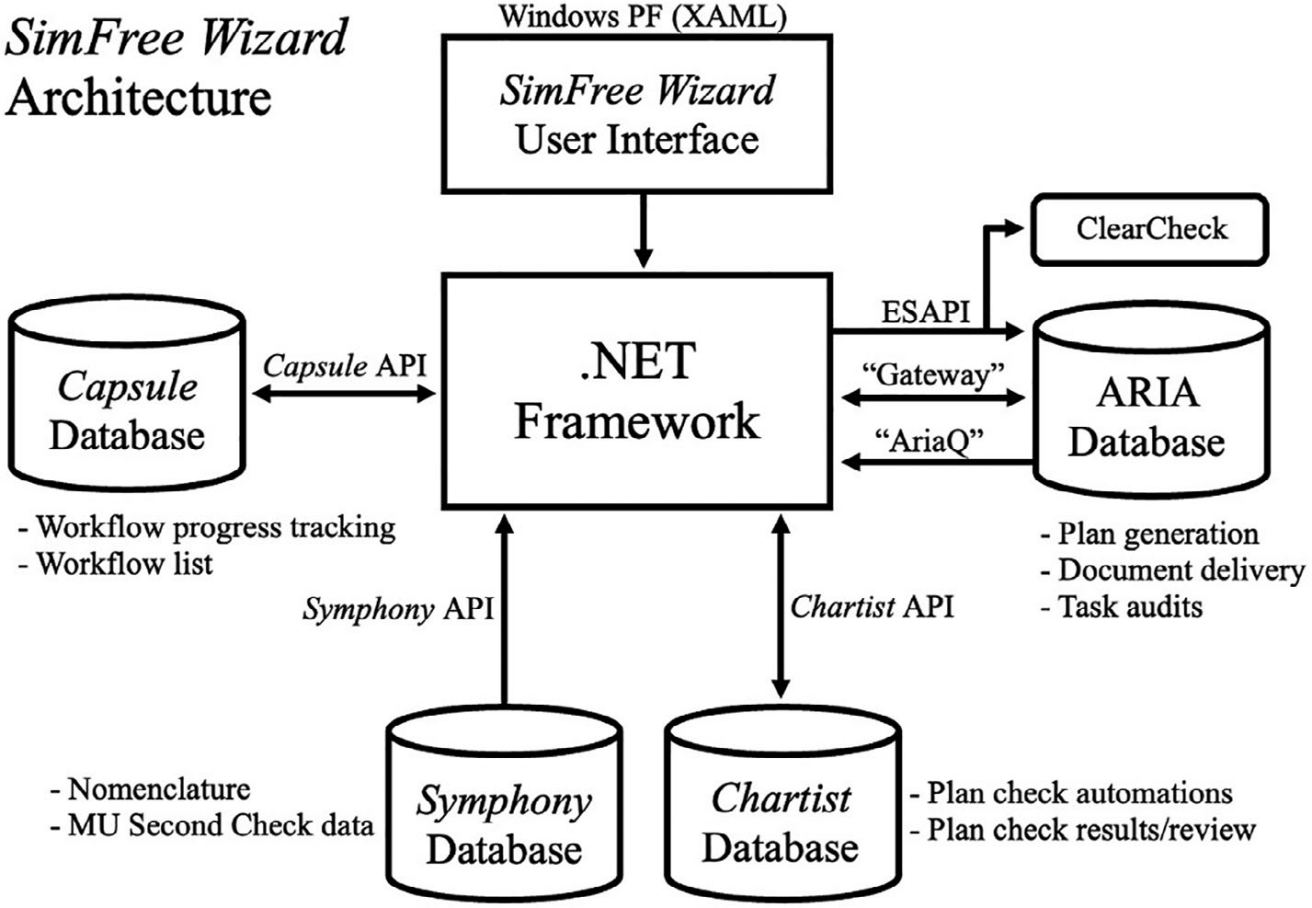
Schematic of the SimFree Wizard data/network architecture. The .NET software development framework formed the foundation for the application, communicating with the applicable databases via various commercial and in‐house scripting application programming interfaces (APIs). The software's UI was developed with Windows Presentation Foundation, an integrated development environment incorporating Extensible Application Markup Language (XAML). APIs, Application programming interfaces; UI, User interface; XAML, Extensible Application Markup Language.

The in‐house platform, christened the *SimFree Wizard* (SFW), was developed with the .NET software development framework through application of the C# programming language and various commercial and in‐house scripting APIs, facilitating data extraction directly from the treatment management system (TMS), treatment planning system (TPS), and internal software repositories. A broad‐level visualization of the SFW architecture is displayed in Figure 1. Scripts generated with the various application programming interface (API) extensions were compiled as a stand‐alone executable to form the coding foundation for the application. Within the software, structured query language (SQL) databases hosted on institutional servers are leveraged to execute rudimentary data management functions and complex queries to convert available raw data into useful and contextual information. The Entity Framework (Microsoft Corporation, Redmond, WA), in support of Language‐Integrated Query (LINQ) technologies, was employed to translate the data in the SQL databases to the C# programming language to facilitate data transfer. Integrated commercial solutions include a vendor‐provided “Web Service” API (“Gateway”) to automatically upload requisite patient documentation into ARIA (Varian Medical Systems, Inc.) via a standard File Transfer Protocol, ARIA querying (“AriaQ”) capabilities to assist in data management, and ClearCheck software (Radformation, New York, NY) to automate the plan evaluation and approval processes. Additionally, the SFW interfaces with several other clinically deployed internal applications when appropriate to perform various needed tasks. The *Capsule* in‐house software was developed in 2021 to serve as the departmental whiteboard, featuring various management tools for physicians, physicists, dosimetrists, and therapists, alongside information on staff coverage, downtime management, and so forth. The *Symphony* platform (released internally in 2023) hosts various scripts to assist physicists and dosimetrists with several important treatment planning and/or plan check functions. Lastly, the *Chartist* software is a semi‐automated, checklist‐driven physics chart checking program leveraging both ESAPI and a direct database connection to ARIA SQL queries. This tool has functioned as the primary platform for all photon‐based initial and final chart checks within this department since its internal deployment in 2020. While a detailed discussion of these supplementary applications is beyond the scope of this report, note that these platforms were developed in a related manner with a similar infrastructure.

The graphical user interface (GUI) was customized via an integrated development environment and framework (Windows Presentation Foundation—Microsoft Corporation) employing Extensible Application Markup Language (XAML), a declarative language based on XML. In general, XAML was utilized to define the visual appearance of the application, while code‐behind logic (code associated with markup) was deployed to implement functionality in response to user interactions. It is anticipated that improvements to the SFW interface will occur over time in response to user feedback, though the general workflow and steps described herein should remain largely unchanged. Note that all code generated during the development of this platform was subject to thorough review and testing by the departmental programming group.

### 2.2 Workflow overview

**FIGURE 2:**
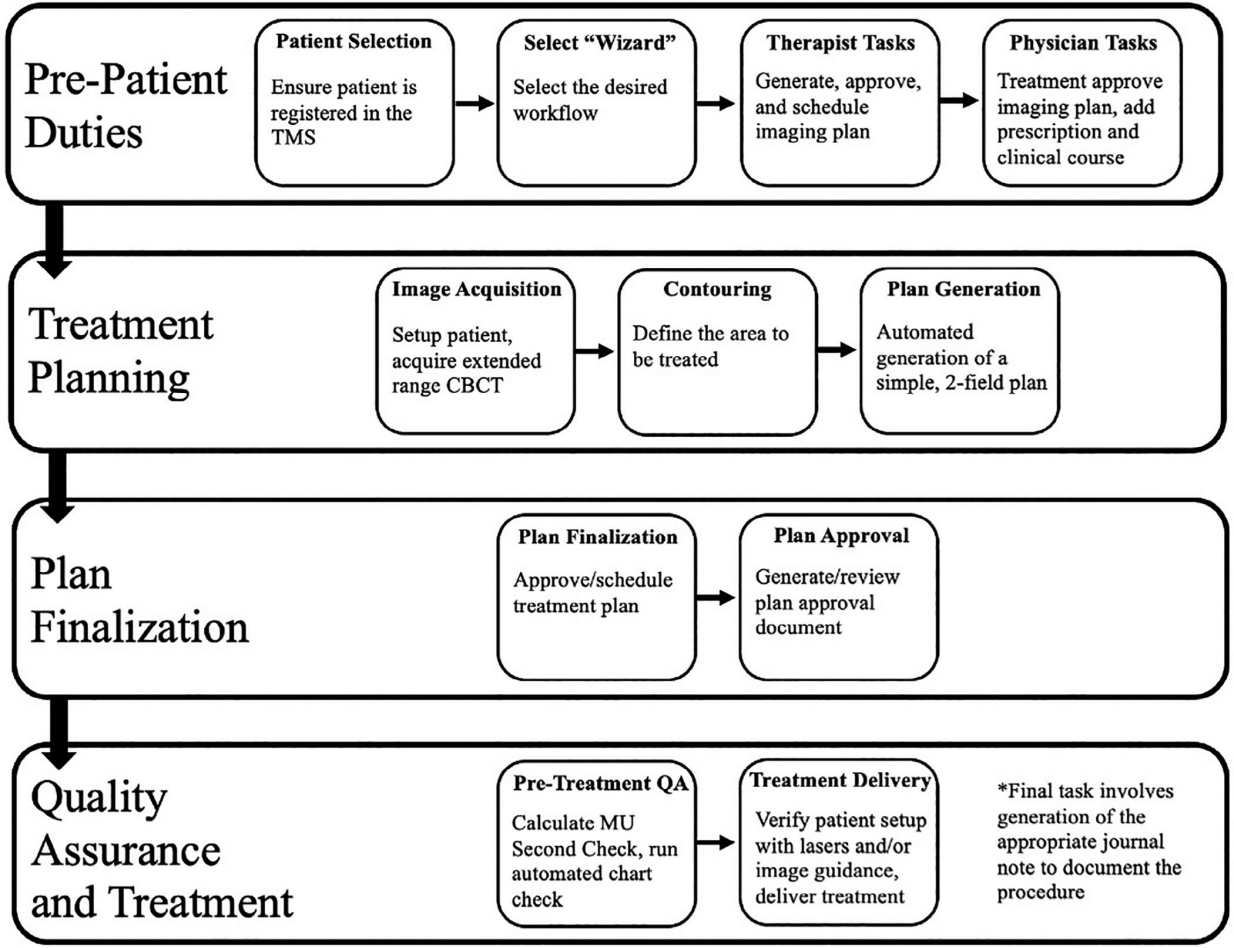
Diagram of the SFW workflow for expedited palliative radiotherapy. Shown for each of the four main “subprocesses” are the requisite “steps” needed for completion. Additionally, the main “tasks” associated with each of the individual steps are provided. SFW, SimFree Wizard.

**FIGURE 3:**
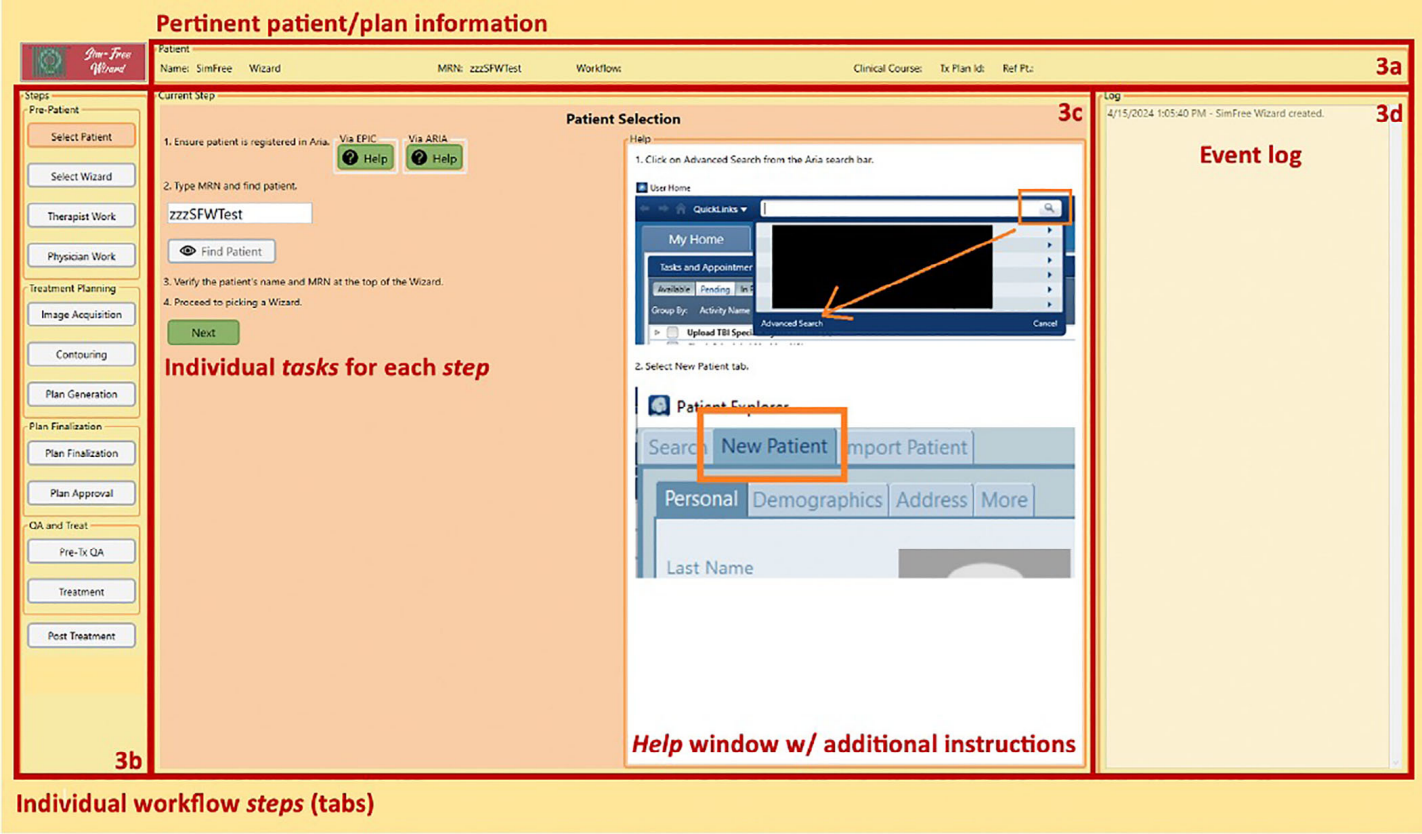
The SimFree Wizard user interface (UI) upon initiation of the application. The uppermost section (a) of the UI displays pertinent patient/plan information. The left‐hand window (b) provides sequential tabs to assist the user in progressing through the individual “steps” of the workflow. Note that tabs are organized based on the “subprocess” to which they correspond. Upon activation of an individual tab, the center window (c) provides piecemeal instructions (i.e., “tasks”) for completing each step, with detailed graphical guidelines for tasks requiring manual execution. Completion of individual tasks is logged and documented on the right‐hand side of the UI (d).UI, User interface.

Though the initial developmental phase has emphasized expediting emergent on‐call and palliative care, the SFW is conceived as a general framework for accelerated CBCT‐based planning workflows that commence with the acquisition of a CBCT image and conclude with treatment in 1‐hour or less. The workflow integrated within the software adheres to all procedural steps in a standard planning workflow, though this process is notably abbreviated in time through the use of full automation when achievable. Provided in Figure 2 is a detailed diagram of the proposed SFW workflow, including the main steps in the process and their associated tasks, while Figure 3 presents a screenshot of the GUI upon initiation of the SFW application for a phantom‐based dry run. As soon as requisite tasks have been executed, pertinent information such as patient name, medical record number (MRN), and treatment type, as well as clinical course, plan, and reference point identifiers, are visible to the user within the uppermost section of the SFW interface (Figure 3a). Progression within the workflow occurs as the user is guided step‐by‐step through the sim‐free RT process, with steps defined by the minimum tasks necessary to safely proceed. For enhanced ease of use and operational simplicity, hierarchical organization of these steps (and their associated tasks) is implemented based on the type of action to be completed and their sequential occurrence in the workflow (as well as which individual is to perform the tasks). All procedural steps are first categorized into comprehensive pre‐patient, treatment planning, plan finalization, or quality assurance and treatment “subprocesses.” Each of these subprocesses is further decomposed into the general “steps” highlighting routine actions to be performed (e.g., patient selection, image acquisition, etc.), which appear as individual tabs on the left‐hand side of the application's user interface (UI) (Figure 3b). Note that each step (delineated by the individual tabs) indicates a traditional point in a standard care path in which data/patient handoff would occur. Upon activation of an individual tab, each step is collapsed into piecemeal instructions for the user on the left‐hand side of the main application window (i.e., “tasks”), while detailed graphical instructions (further directions with screenshots when necessary) are provided on the right‐hand side of the window for active tasks mandating manual execution (Figure 3c). The tasks within the tabs necessitate the user's selection of a checkbox upon completion to initiate an automated audit (when possible) to verify satisfactory execution of the task prior to proceeding. If the user has failed to perform the task as necessary, a pop‐up window will alert the user to this discrepancy. The user can also verify successful completion of a task by noting if it appears with the appropriate time‐stamp on the event log visible on the right‐hand side of the UI (Figure 3d). Given the interdependent nature of later tasks on the execution of earlier ones, progression to the next task within a tab (and from tab to tab) cannot occur until the prior task has been completed, forcing the user to advance sequentially through the workflow. Upon fulfillment of all requisite tasks for a given step, the application automatically transitions to the subsequent tab, and the completed tab is grayed out to prohibit user regression to previous steps.

Of note, all CBCT images utilized for plan generation within this platform are acquired via a novel image reconstruction infrastructure, iterative CBCT, recently integrated on conventional TrueBeam linear accelerators (Varian Medical Systems, Inc.). Details pertaining to this advanced imaging solution have been provided in the literature,^30^, ^31^ while recent studies have demonstrated a noticeable improvement in image quality and an increased dose calculation accuracy relative to previous CBCT systems.^20^, ^21^, ^32^ Users are instructed to utilize the standard *Pelvis* scanning protocol (125 kVp, 1080 mAs, 2.0 mm slice thickness, 46.5 cm field‐of‐view [FOV]) as this setting applies the highest available preset mAs value to mitigate image noise and maximize image quality (though any scanning protocol can be selected at the treatment unit). A previous study established a CBCT‐based dose calculation accuracy within 0.5% for target volumes and 1.0% for organs‐at‐risk for this scanning protocol (with application of the standard CT calibration curve).^21^ Therefore, the performance of this onboard imaging system has been deemed more than sufficient for the intended uses within the SFW tool. Additionally, the Anisotropic Analytical Algorithm with the Eclipse TPS (AAA—v15.6.06, Varian Medical Systems, Inc.) model is utilized to calculate volume dose with a dose grid resolution of 0.25 mm, though it is anticipated that the linear Boltzmann‐based AcurosXB algorithm (Varian Medical Systems, Inc.) will be integrated clinically within the coming year (and will serve as the main calculation model at that time). Furthermore, to allow for an increased longitudinal scan extent and to ensure appropriate scattering conditions are present for the plan generation and dose calculation task, guidance for acquiring an extended range CBCT is provided for the user (while for standard CBCT images the longitudinal scan extent is ∼16 cm, with an extended range scan this can be increased to ∼32 cm). Additionally, a larger superior‐inferior scan extent allows for the inclusion of a greater number of vertebral bodies to ensure accurate localization of the desired treatment area.

For a detailed workflow description of the four main subprocesses, including the requisite steps and associated tasks, the reader is referred to the Supplementary Materials document (Section S.I.).

### 2.3 Development and testing

#### 2.3.1 Process timing and usability testing

Upon development of the SFW, formal dry runs were conducted to further refine the platform and its associated workflow and to quantify relevant timing metrics. These sessions also served the critical role of training individuals in the application of this software. Of note, the phantom utilized to conduct the testing was a standard anthropomorphic lung and chest phantom (RS‐310, Radiology Support Devices, Inc., Long Beach, CA) constructed from materials designed to have similar mass densities and attenuation coefficients to natural bone and soft tissues. To objectively assess the duration of the proposed treatment strategy for expedited palliative spine RT, timing data from 10 phantom‐based dry runs with various operators (both therapists and physicians) was acquired once the software was fully operational. While no formal timing data was available for either emergent UTM‐based or standard single‐day palliative treatments (denoted sim‐and‐treat at this institution), users were asked to estimate the time requirements for these processes for the purpose of comparison. Throughout the testing phase, all users (including physicians) were also asked to provide constructive feedback in addition to responses to the following questions regarding use of the platform for expedited palliative spine RT—“How clear is the provided direction/guidance in the platform for the completion of this process?” (1 = unclear, 5 = very clear), “How easy is this process to complete?” (1 = difficult, 5 = very easy), and “How confident are you that this process was performed correctly/safely?” (1 = not confident, 5 = very confident). To permit further comparative analysis between the developed platform and the previously employed UTM solution, recent users of UTM were solicited to assess the ease of use of that process by responding to the same aforementioned questions. These responses will be utilized to improve the training resources and overall usability of the SFW platform and to address any safety concerns voiced by the users.

#### 2.3.2 Second check software validation

Following plan generation and prior to treatment initiation, an independent monitor unit (MU) second check is performed within the “Pre‐Treatment QA” tab via an in‐house function incorporated into the SFW. Dose differences between the expected and calculated values should generally be less than ± 5.0%. For simple beam arrangements (as in the “AP/PA Spine” workflow), the second check is a tissue phantom ratio (TPR)‐based point‐dose calculation (at isocenter) incorporating appropriate in‐air collimator scatter (*S_C_*) and phantom scatter (*S_P_*) factors. For each beam, the anticipated point‐dose is calculated according to Equation (1):

(1) $$\mathrm{Dos}e_{\mathrm{calc}}=\frac{\mathrm{Dos}e_{\mathrm{expected}}}{\mathrm{TMR}\;\times S_{C}\times S_{P}\times \mathrm{InvSq}}$$

where Dose_expected_ for the simple two‐beam arrangement is half of the prescribed dose, and the InvSq correction factor accounts for the dose fall‐off variation in the position/depth of the calculation point relative to the calibration setup conditions. The *TPR* value, based on the equivalent square field size and effective depth as reported by ESAPI (the effective depth incorporates a ray‐based depth correction accounting for inhomogeneities present in the imaging dataset), is specific to each beam and is determined via a lookup table within the *Symphony* in‐house software. All TPR data was derived from an independently collected set of depth dose curves. Furthermore, the *S_C_* and *S_P_* values are sourced from the *Symphony* database as well. Of note, *S_C_* is a function of the effective field size (not the equivalent square field size), a key demarcation for future workflows in which the multi‐leaf collimators (MLCs) will be utilized.

To validate this in‐house MU second check software developed for the SFW tool, the agreement in point‐dose calculations at midline between the internal solution and the Eclipse TPS was evaluated for a variety of simple treatment fields with assorted energies, field sizes, depths, inhomogeneities, and so forth, for both slab phantom‐ and anthropomorphic thorax phantom‐based geometries. Additionally, representative fields were delivered to CBCT datasets of patients previously treated at this institution to assess the algorithm's performance on actual patient data. For a detailed description of the validation testing, the reader is referred to the Supplementary Materials document (Section S.II.).

Of note, all plans are also automatically exported for analysis by a commercial second‐check software (Mobius3D, Varian Medical Systems, Inc.) utilized for all other photon‐based plans in the clinic (with a button available within the SFW to launch the web browser if needed). However, as this solution often takes at least several minutes to complete and return results, it is only intended to be a redundancy check for simple treatment scenarios should the in‐house results be outside of the expected tolerance. For application of the SFW in more complex, curative scenarios (as anticipated in the future), this software will likely serve as the primary means of secondary verification.

### 2.4 Clinical case

A 76‐year‐old female with diagnosis C79.51 (secondary malignant neoplasm of bone) presented in the department in August 2024. Upon consultation, the physician opted for palliative RT via the in‐house SFW platform. Spinal metastases involving the L4 to S1 vertebral bodies were treated with 2000 cGy in five fractions (400 cGy/fraction) via 18 MV parallel‐opposed AP/PA beams. Treatment occurred within standard working hours, with two radiation therapists and a physician overseeing treatment planning and delivery. Though not required within the workflow, a physicist was also present to provide technical support as necessary.

## 3 RESULTS

### 3.1 Process timing

**FIGURE 4:**
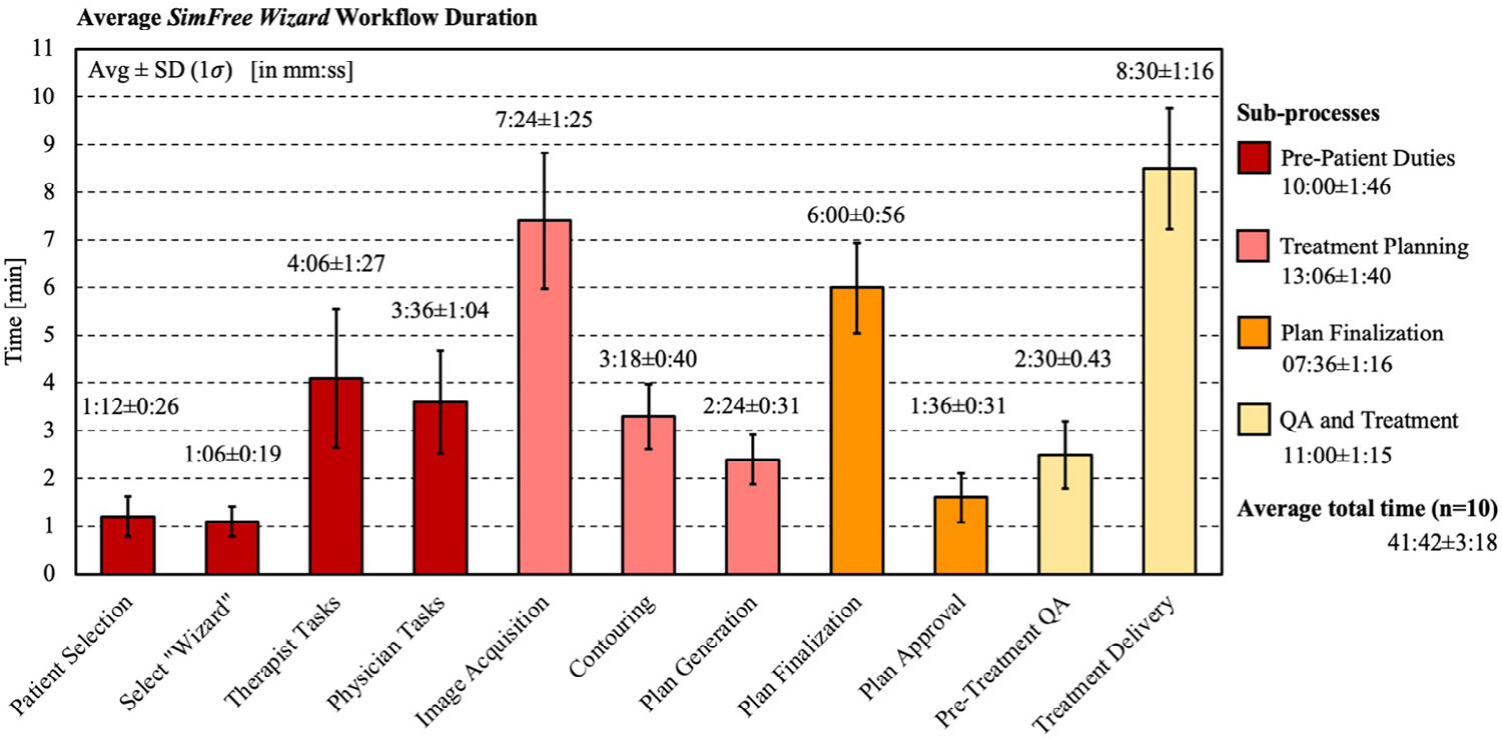
Average timing metrics for each individual *SimFree Wizard* workflow step for 10 palliative spine phantom‐based dry runs. Listed on the right‐hand side of the graph are the average timing metrics for each aggregate subprocess and the average total time required to execute the entire workflow. Average timing results are reported to one standard deviation.

Detailed timing data pertaining to the various workflow steps for 10 phantom‐based dry runs are presented in Table 1, while average values for these metrics are graphically depicted in Figure 4. The mean time required to execute the entire SFW workflow for palliative spine RT was 41:42 ± 3:18 [mm:ss] (min ∼37 min, max ∼46 min), with the image acquisition (average of 07:24 ± 01:25, min ∼5 min, max ∼10 min) and treatment delivery (average of 08:30 ± 01:16, min ∼6 min, max ∼10 min) steps necessitating the longest duration. Of the four pertinent subprocesses, the combined treatment planning steps (image acquisition, contouring, and plan generation) were the lengthiest on average, requiring a mean time of 13:06 ± 01:40 (min ∼11 min, max ∼16 min) across the 10 trial runs. Notably, all trial runs were completed in under 50 min, and these timing metrics were ascertained across tests performed by an assortment of users with varying levels of training on the platform, highlighting the robustness of this temporal framework (range ∼10 min) for various users due to the integrated automation. Importantly, given that all pre‐patient duties can be performed prior to the patient's placement on the couch (and thus prior to patient arrival for standard palliative cases), the steps in the workflow requiring the patient to be present were completed in 31:42 ± 02:48 on average (min ∼29 min, max ∼36 min).

**TABLE 1:** Process timing data for ten palliative spine phantom‐based dry runs with the *SimFree Wizard* platform.

Overall workflow duration		Individual step‐by‐step duration (*n* = 10)			
Trial run number	Total time (min)	Step	Average (mm:ss)	Maximum (min)	Minimum (min)
1	46	Patient selection	1:12 ± 0:26	2	1
2	43	Select “Wizard”	1:06 ± 0:19	2	1
3	38	Therapist tasks	4:06 ± 1:27	6	2
4	39	Physician tasks	3:36 ± 1:04	5	2
5	45	Image acquisition	7:24 ± 1:25	10	5
6	46	Contouring	3:18 ± 0:40	4	2
7	40	Plan generation	2:24 ± 0:31	3	2
8	37	Plan finalization	6:00 ± 0:56	7	5
9	44	Plan approval	1:36 ± 0:31	2	1
10	46	Pre‐treatment QA	2:30 ± 0:43	4	2
**Average**:	**41:42 ± 3:18**	Treatment delivery	8:30 ± 1:16	10	6

*Note*: Listed is the overall workflow duration for each of the ten trial runs and the average, maximum, and minimum timing metrics for the individual process steps. Average timing results are reported to one standard deviation, while all other results are rounded to the nearest minute.

In contrast, when asked about temporal metrics for emergent UTM‐based treatments, feedback indicates that the workflow duration has historically been anywhere from 1–3 hours contingent on the therapist's and physician's familiarity and comfort with the UTM software (as well as the condition of the patient). One physician even noted a previous treatment session with UTM surpassing 5 hours in duration. While the SFW software can automatically generate satisfactory multi‐field CBCT‐based 3D treatment plans in a matter of seconds, manual single‐field plan generation on 2D radiographs with UTM requires ∼10 min or more, depending on the user. Though the time required to execute the SFW workflow will also vary somewhat with therapist and physician understanding and comfort levels, the extent of this variation was demonstrated to be much less in the presented trial runs given the small range (∼10 min) in the total process duration across a variety of users. Additionally, completing standard palliative spine RT treatments with the SFW platform is expected to yield discernible time and resource savings compared to the single‐day sim‐and‐treat process currently utilized at this institution. For this workflow, patients are first required to undergo a conventional CT simulation (preferably in the morning), necessitating anywhere from 30 min to 1 hour on one of the clinic's CT units. Consensus reports from the photon‐based dosimetry team at this institution indicate that, following image acquisition, it takes approximately 15–20 min to import these images into the TPS and prepare them for the physician, 30 min to 1 hour for the physician to provide the prescription and delineate the intended treatment area (varying based on physician availability in the clinic), 30 min to setup and plan standard AP/PA palliative spine RT treatments, 15 min to 1 hour for physician review and approval, and 20 to 30 min for plan finalization and documentation. A physicist must then perform a chart check prior to treatment approval. As such, the duration required to execute the standard sim‐and‐treat process at this institution is somewhat variable, often requiring nearly one full working day for completion.

### 3.2 Usability testing

**FIGURE 5:**
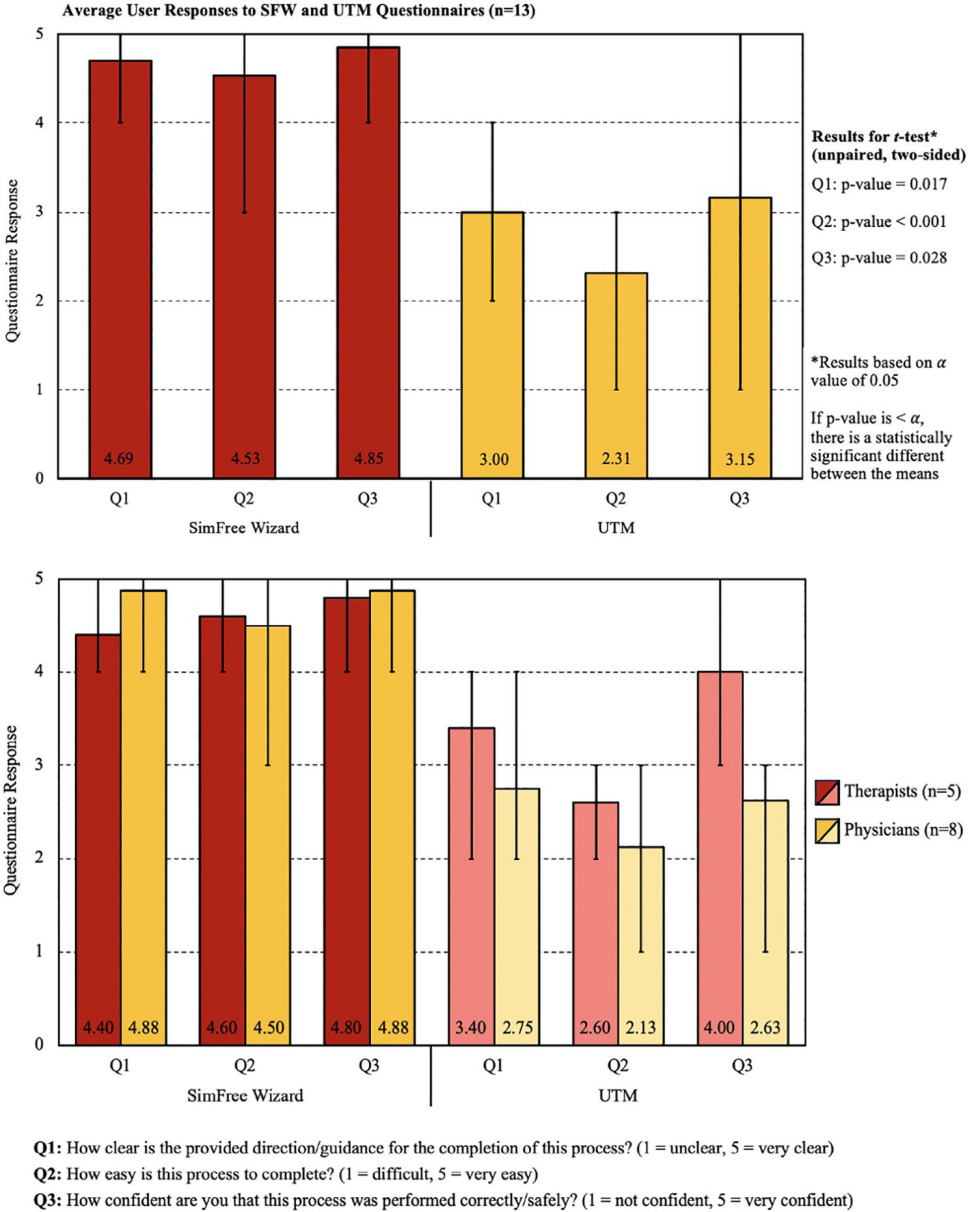
Average user responses for each question on the *SimFree Wizard* (SFW) and *Unplanned Treatment Mode* (UTM) questionnaires for (a) all users (*n* = 13) and (b) therapist (*n* = 5) and physician (*n* = 8) users independently. The mean numerical values are provided above each question number, while the error bars indicate the range of provided responses. SFW, SimFree Wizard; UTM, Unplanned Treatment Mode.

Average responses for all users (*n* = 13) who participated in the 10 trial runs are depicted in Figure 5a, while the distribution of these responses between therapists (*n* = 5) and physicians (*n* = 8) is illustrated in Figure 5b. Overall, users reported improved usability for SFW relative to UTM, evident by average responses for SFW of: Q1: 4.69 (range 4–5), Q2: 4.53 (range 3–5), and Q3: 4.85 (range 4–5) compared to Q1: 3.00 (range 2–4), Q2: 2.31 (range 1–3), and Q3: 3.15 (range 1–5) for UTM (Figure 5a). Statistical analysis via an unpaired, two‐sided *t*‐test (with *α* = 0.05) confirmed the significance of these comparative results, with calculated *p*‐values of 0.017 (Q1), < 0.001 (Q2), and 0.028 (Q3) between the mean responses for the two platforms. Importantly, the largest variation in feedback between SFW and UTM was observed for Q2 (ease of use), likely owing to the incorporated automations in SFW and a platform designed to explicitly direct the user step‐by‐step through the process.

Of note, responses also varied slightly between therapist (*n* = 5) and physician (*n* = 8) users involved in this testing phase (Figure 5b), with larger discrepancies observed for the UTM questionnaire. On average, physicians expressed slightly lower levels of confidence and ease of use for UTM when compared to their therapist counterparts (while average physician responses for the SFW questionnaire were slightly higher for Q1 and Q3). Furthermore, the variability in individual responses (the error bars on the graph denote this range) was lessened for SFW when compared to UTM. Provided comments indicate that, for UTM, user responses were highly correlated with the level of training and experience with the platform, whereas for SFW, users offered comparable responses regardless of their training and familiarity with the tool. Additional comments that noted desired changes to the UI or further clarification/instructions for various workflow steps have been or will be incorporated in future iterations of this platform.

### 3.3 Monitor unit (MU) second check software validation

**FIGURE 6:**
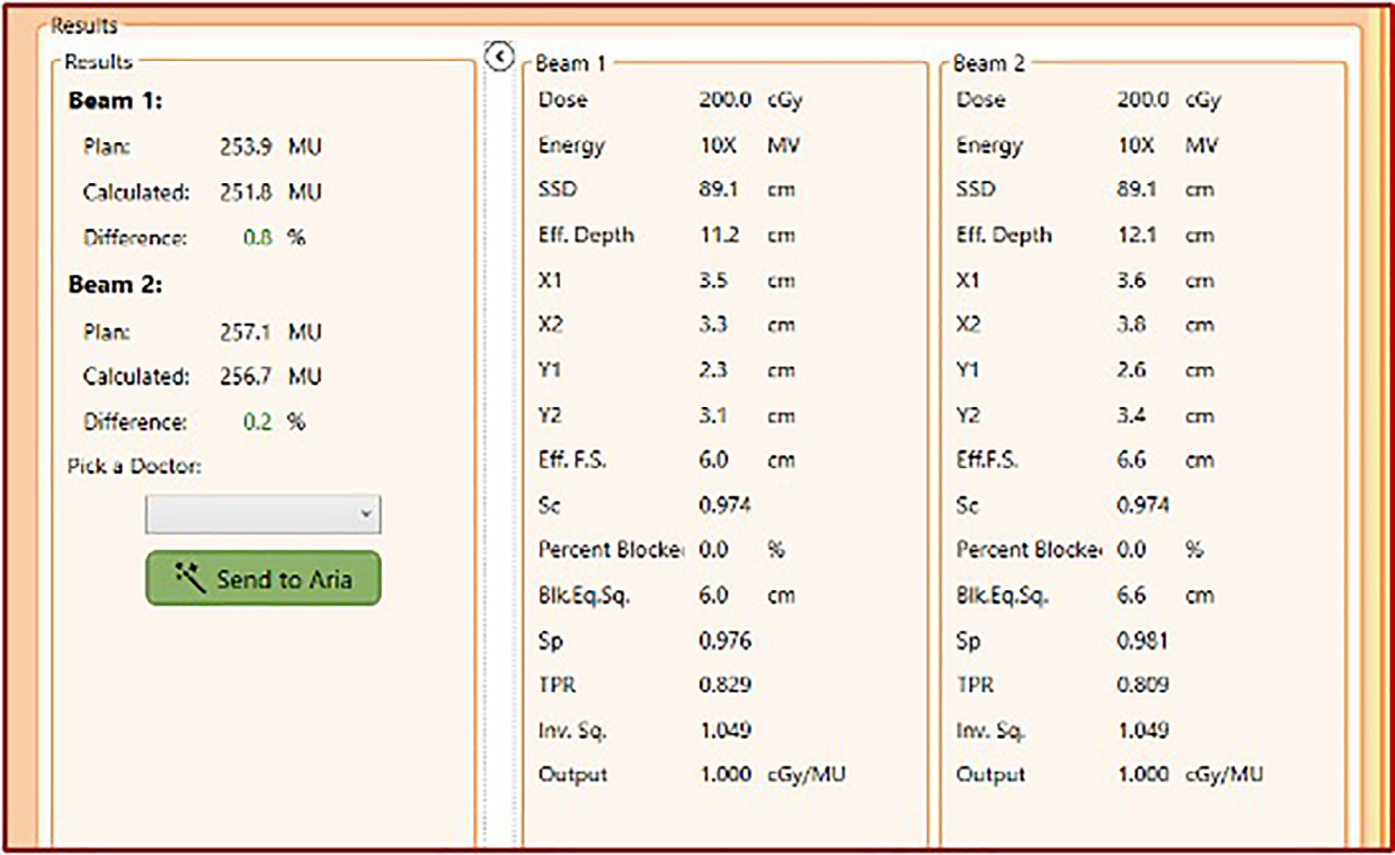
Monitor Unit (MU) second check results for one of the phantom‐based dry runs. The 10 MV plan was generated with a prescription of 400 cGy for 5 fractions to a total of 2000 cGy. The results on the left‐hand side show the percent difference in the number of MUs computed by the treatment planning system (“plan”) and the second check software algorithm (“calculated”) for each of the beams. Factors utilized in the MU calculation (as provided in Equation (1)) specific to each beam are displayed for the user on the right‐hand side. Note that a drop‐down menu is provided, allowing the user to select a physician and then export a signed secondary verification document to the patient's documents tab in ARIA. MU, Monitor unit.

Detailed results from the validation testing can be found in the Supplementary Materials document (Section S.II.). For all validation calculations conducted on virtual solid water or heterogeneous slab phantom geometries generated within the TPS, all discrepancies between the TPS calculations and the secondary verification software were within ± 3.0%, with average values for each energy generally within ± 1.0%. All secondary verification results for the 10 dry runs in this study were within the expected tolerance of ± 5.0%, with 65% of fields (13/20) exhibiting deviations less than ± 1.0%. An example of the second check results displayed within the UI for one of the trial runs is provided in Figure 6. The validation results on the clinical patient datasets exhibited variability, with the location of the calculation point seeming to have the most substantial influence on the accuracy of the computed values. The magnitude of variation displayed minimal correlation with the selected energy or field size; however, it was observed that for fields selected in the mid‐ to upper‐thoracic spine region, slightly larger discrepancies were calculated for all energies due to the increased presence of lung tissue within the treated area, even if the calculation points remained in soft tissue. In general, the number of MUs calculated by the second check software was marginally lower than that calculated by the TPS.

### 3.4 Clinical case

**FIGURE 7:**
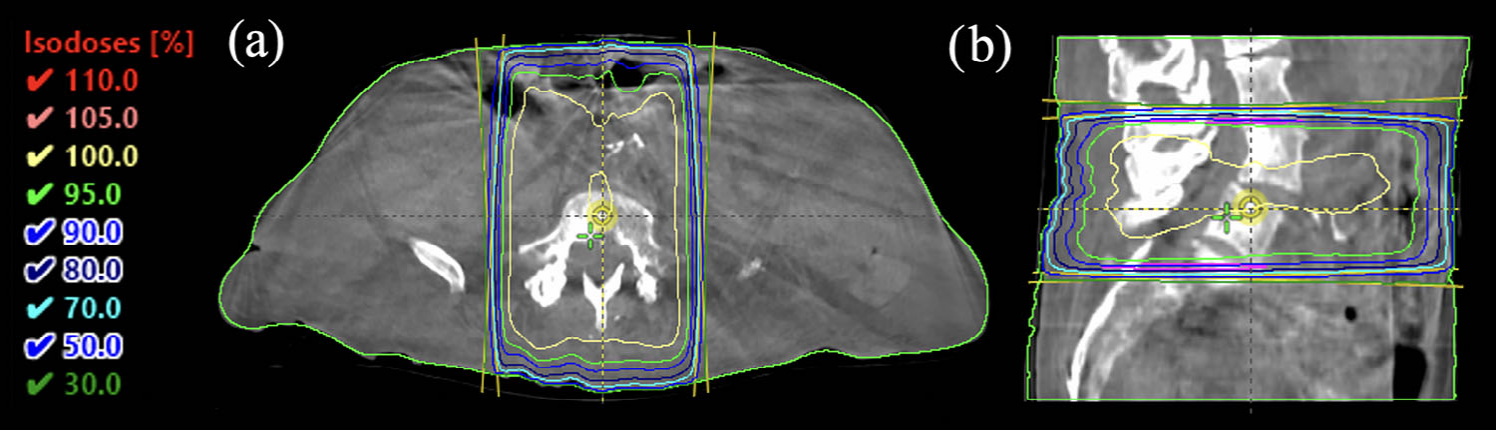
Dose distribution for an AP/PA treatment plan for spinal metastases delivered with the developed platform in the clinic. Shown are (a) axial and (b) sagittal slices for 18 MV beams with a prescription of 2000 cGy in five fractions (400 cGy/fraction) delivered to vertebral bodies L4 through S1.

Palliative spine RT for secondary bony metastases was successfully administered using the SFW platform. A single CBCT image was acquired for plan generation (given the extent and location of the target area, an extended‐range CBCT was not required). For setup verification prior to treatment administration, an MV portal image was captured from the beam's eye view. The delivered dose distribution is displayed in Figure 7.

Pre‐patient duties were completed in 06:30 [mm:ss], with most tasks finalized prior to patient arrival. The treatment planning steps (beginning with acquisition of the CBCT image for plan generation) were completed in 23:16. While there was initially an issue with image acquisition (not due to the in‐house software) that delayed progress through the workflow, this issue was resolved upon reacquisition of the CBCT image for planning. The plan finalization steps required 05:27, while pre‐treatment QA and treatment delivery were executed in 08:26. In total, completion of all procedural steps required ∼44 min. Additionally, the initial patient setup process (prior to image acquisition for plan generation) required ∼9 min. Overall, the patient was in and out of the treatment room in less than 50 min. No issues were reported with the use of the developed software.

## 4 DISCUSSION

This manuscript outlines the development of an in‐house, semi‐automated, sim‐free RT platform engineered to facilitate accelerated CBCT‐based treatment workflows for the administration of expedited palliative RT. This technique has the potential to enhance patient safety (particularly in emergent scenarios) while mitigating patient discomfort, increasing departmental efficiency, and thereby improving patient access to palliative care. In contrast to conventional 2D clinical planning and setup protocols as commonly applied in palliative settings, the implementation of CBCT affords enhanced 3D anatomic information, improving dosimetric accuracy as well as precision in the delineation of the desired treatment area. Furthermore, this approach allows for 3D documentation of the RT plan, enabling efficient calculation of composite quantitative spatial dose information should additional palliative RT regimens be required in the future. As the development and initial testing phases of this software have concluded as detailed in this study, the SFW has recently received approval for clinical release and implementation by the department's clinical practice committee.

Analysis of the temporal metrics highlights the potential reduction in procedural duration achievable through application of the proposed approach. Prior user feedback for UTM noted that this process generally requires 1–3 hours depending on the complexity of the patient case and the therapists’ level of proficiency and training with the platform. Likewise, the expedited sim‐and‐treat process currently employed at this institution typically demands nearly a full working day for completion. Consequently, patients undergoing such treatments are required to visit the clinic multiple times over the course of a single day should they elect to leave following the CT simulation or must endured prolonged wait times within the clinic (on the order of hours) prior to their treatment, both of which are inconvenient for the patient and personnel in the department. Moreover, the delivery of these plans often necessitates therapists, physicians, and physicists to stay after‐hours given the uncertainty as to when exactly the plan will be ready for treatment (since concurrent clinical duties must be fulfilled in the meantime, it is difficult to schedule time on one of the machines during busy clinic hours). However, the SFW automates many of the requisite tasks, and the acquisition of a standard CT simulation image is no longer required for patients in this workflow. Thus, this proposed framework allows for enhanced resource allocation efficiency and enables the ability to now complete the entire sim‐and‐treat process in approximately 1 hour or less. Such treatments can therefore be scheduled during normal clinic hours.

However, it is important to acknowledge that timing for patient treatments with SFW, at least initially, will likely exceed the average phantom‐based metrics reported in this study, as observed within the presented clinical case (total time for entire workflow completion ∼53 min). The actual time required for completion of this process in the clinic is contingent upon several critical factors, including the complexity of the case and the patient's health status in addition to patient compliance with the provided instructions. As an aside, in scenarios in which the patient experiences substantial discomfort while lying in the imaging and treatment setup position, the proposed workflow permits temporary removal from the couch following image acquisition while the plan is being generated and prepared for delivery. Extra user caution will also be exercised when proceeding through this workflow during the preliminary clinical implementation phase. Despite these considerations, it remains the expectation that this process can be completed within a 1‐hour window for a vast majority of the anticipated patient cases.

In addition to reducing workflow duration, feedback derived from the provided questionnaires regarding platform usability demonstrated a unanimous preference for SFW over UTM. In general, therapists and physicians offered similar responses for SFW regardless of their level of training or experience with the platform, further highlighting the overall enhanced usability of SFW compared to UTM. In particular, therapist feedback noted that additional comfort is afforded by the potential of improved physics support available with SFW (as this program was developed by physicists). For after‐hours, emergent treatments with UTM, there is typically limited or no physics assistance available unless absolutely necessary. Importantly, despite the limited physics support and generally reduced ratings for UTM (particularly for Q3 regarding confidence in this process), there have been no recorded instances of misadministration or treatment error when utilizing this platform in the years preceding the development of SFW. However, the objective of introducing the SFW solution was to provide a more favorable user experience when performing expedited palliative RT, and the collected user feedback unanimously indicates that this goal has been successfully realized.

Adjacent to the development of the SFW platform, a dedicated secondary verification software was engineered for the expeditious validation of calculated MUs within the platform. While deviations beyond ± 5.0% were observed in several instances, a critical aim of the validation process, as previously articulated, was to determine the limitations of this algorithm and acquire insight on expected values should verification calculations occur for “nonstandard” cases or those in which relatively large amounts of heterogeneities are present within the treatment fields. This derived knowledge enables the education of therapists regarding the range of expected/acceptable values for such scenarios. Additionally, the actual MUs delivered within the treatment plan are calculated by a rigorously commissioned TPS and are no longer reliant on simple hand calculations (prone to user error) as is common practice for palliative RT. In general, the reported results pertaining to this refined validation process demonstrated good agreement with the TPS for a variety of energies and field dimensions and provide confidence that the in‐house MU secondary verification software is accurate for palliative spine RT as proposed and for other simple cases in which the dose calculation occurs in a predominantly homogenous medium (e.g., whole brain RT). As noted, in instances where results outside of the recommended tolerance occur with the simple algorithm (e.g., for palliative lung RT), results from the commercial secondary verification software (Mobius3D) can be considered. Likewise, as more complex treatment techniques are introduced within the platform, Mobius3D will be incorporated for the primary second check process as previously stated.

The preliminary development phase has concentrated on expediting palliative spine RT, as recent clinical practice patterns at this institution indicate that nearly all emergent, on‐call cases in recent years have been of this type. The initial clinical release of this platform will be restricted to simple parallel‐opposed AP/PA treatment fields with equal beam weighting until the software has undergone further validation through several patient cases in a clinical environment and it has been verified that there are no unforeseen complications in such scenarios. Given that these workflow steps and automated processes have been refined during the development stage, integrating additional treatment and delivery techniques within this platform moving forward is anticipated to be a fairly trivial process requiring minimal modifications to the workflow. Additionally, per physician request, the second clinical iteration of this software is expected to allow for single PA treatment fields in addition to refined AP/PA fields with a 2:1 weighting ratio favoring the posterior beam. As is, the existing code framework also supports simple palliative treatments with parallel‐opposed AP/PA beams for any anatomical region (though available nomenclature options within the software would need to be updated to be applicable to additional sites). Moving forward, the next treatment site/technique to be included will be whole brain RT utilizing laterally‐opposed fields. Note that this technique will likely demand increased physician involvement during the plan setup process due to the incorporation of MLCs for ocular/lens shielding.

While the simple treatment techniques as discussed are effective in streamlining delivery, a notable limitation of the current approach is that such plans result in relatively suboptimal conformality with elevated radiation doses to adjacent organs. In palliative spinal bone irradiation, AP/PA field plans have been demonstrated to accomplish the International Commission on Radiation Units and Measurements (ICRU) Report 50 (*Prescribing, Recording, and Reporting Photon Beam Therapy*) recommendations for the intended dose ranges, achieving a homogenous distribution with reasonable exposure to critical OARs.^33^ Consequently, this approach remains markedly superior to conventional 2D single posterior field RT as traditionally employed with UTM (which do not fulfill ICRU Report 50 recommendations). Nonetheless, as patients with osseous metastases continue to experience prolonged survival, the probability of manifesting delayed complications has increased, indicating a potential need for more conformal palliative plans for certain patient populations.^34^ Of note, the software delineated within this study has been engineered in such a way as to facilitate the inclusion of more sophisticated, curative treatment techniques with minimal perturbation to the overall workflow, and substantial amounts of code and scripting needed to implement workflows for more conformal treatments have already been under development in conjunction with previous internal studies.^35^, ^36^ Though outside the scope of this initial evaluation, further refinements to the platform and workflows to support more complex, curative approaches will be the subject of future studies.

Lastly, it should be acknowledged that commercial solutions are available to facilitate expedited CBCT‐based planning and delivery procedures in an analogous manner. While the in‐house tool utilized ESAPI in the development of the semi‐automated workflow as presented, platforms are now available that provide the ability to complete similar expedited workflows without the need for scripting capabilities. For example, a recent phantom study demonstrated the feasibility of sim‐free palliative RT with the Ethos platform (Varian Medical Systems, Inc.) via a workaround of the commercial adaptive radiotherapy workflow.^37^ However, the procurement of such a platform represents a significant capital investment for any clinic. At the time of writing, fewer than 50 Ethos units were clinically operational throughout the United States (mainly confined to large academic institutions), with less than 200 units in use worldwide. On the other hand, the number of TrueBeam systems in clinics exceeds these figures by at least an order of magnitude. Therefore, in developing the SFW, resources more commonly available across the broader radiation oncology community were leveraged to promote the potential for more accessible and widespread use of such expedited workflows. Any clinic equipped with a TrueBeam system (or a similar conventional treatment platform) with sufficient imaging capabilities can utilize the API associated with the commercial TPS (e.g., Eclipse) to develop a process akin to that outlined in this study, obviating the need for a dedicated platform or commercial solution such as Ethos for this purpose. The proposed SFW solution also necessitates little to no dosimetry involvement (and limited physics involvement for simple palliative scenarios), a potentially significant benefit to clinics already constrained by limited resources. Furthermore, the workflow described in the referenced study^37^ still requires acquisition of a preliminary image to be imported into Ethos as the reference scan for the initial offline preplan. In contrast, only a single CBCT acquisition is required with SFW. As stated in the preceding paragraph, refinements to the SFW are currently in progress to enable more complex, curative approaches that are more easily achieved with the Ethos planning software at the present time. Nonetheless, the development of the SFW platform and its current and potential future use cases described herein present an exciting advancement in accelerated CBCT‐based planning capabilities with conventional treatment systems.

## 5 CONCLUSION

This report delineates the successful development of an in‐house clinical platform for accelerated CBCT‐based treatment planning and administration, enabling completion of the entire RT workflow within approximately 1 hour or less for expedited palliative and emergent on‐call scenarios requiring simple field geometries. This tool leverages both internal clinical programming applications and commercial software solutions to achieve this objective. Phantom‐based simulations have highlighted the notable time savings of this proposed technique when compared with the existing expedited palliative RT procedures presently employed at this institution, and feedback from both therapists and physicians provides evidence that this solution enhances the overall user experience relative to the current approaches. Results from one of the first clinical cases treated via application of the SFW were presented as well to provide evidence of successful integration in the clinic. The implementation of the described platform offers an expeditious approach to palliative RT that limits the overall burden on patients and their caregivers, thereby improving patient access to palliative care. Additionally, it is presumed that ongoing developments will serve to broaden the clinical applicability of this technique across multiple disease sites, aligning with the ever‐evolving paradigm shift in the field of RT towards integrated CBCT‐based planning and delivery solutions.

## AUTHOR CONTRIBUTIONS

**Riley C. Tegtmeier**: Conceptualization; data collection/curation; formal data analysis; investigation; methodology; writing—original draft. **Edward L. Clouser**: Conceptualization; scripting and automation; data extraction; data curation; investigation; methodology; writing—review and editing. **Quan Chen**: Conceptualization; supervision; writing—review and editing. **Courtney R. Buckey**: Conceptualization; supervision; writing—review and editing. **Suzanne J. Chungbin**: Conceptualization; platform testing; investigation; writing—review and editing. **Christopher J. Kutyreff**: Conceptualization; data curation; writing—review and editing. **Jose S. Aguilar**: Conceptualization; platform testing; investigation; writing—review and editing. **Amber L. Labbe**: Conceptualization; platform testing; investigation; writing—review and editing. **Brooke L. Horning**: Conceptualization; platform testing; investigation; writing—review and editing. **William G. Rule**: Conceptualization; supervision; data review; writing—review and editing. **Sujay A. Vora**: Conceptualization; supervision; data review; writing—review and editing. **Yi Rong**: Conceptualization; supervision; investigation; methodology; writing—primary review and editing.

## CONFLICT OF INTEREST STATEMENT

There are no conflicts of interest to disclose.

## FUNDING STATEMENT

There are no sources of financial support to disclose.

## Supporting information



Supporting Information

## Data Availability

Research data that support the findings of this article will be shared upon reasonable request to the corresponding author.
