# Supplementary material for: Development of an automated CBCT‐based simulation‐free platform for expedited palliative radiotherapy on a conventional linear accelerator
Source: J Appl Clin Med Phys. 2024 Dec 23;26(4):e14612. doi: 10.1002/acm2.14612 (PMC11969108; doi:10.1002/acm2.14612)
Supplement: Supplementary file 1 — Supporting Information [file ACM2-26-e14612-s001.pdf]

SUPPLEMENTARY MATERIAL FOR

"Development of an Automated CBCT-Based Simulation-Free Platform for Expedited Palliative Radiotherapy on a Conventional Linear Accelerator"

S.I. WORKFLOW DESCRIPTION

S.I.A. Pre-Patient Subprocess

The "Patient Selection" step initiates the SimFree Wizard (SFW) workflow. To complete this step, the user (referring to one of the on-call therapists unless deliberately identified as the on-call physician) must first confirm the patient is registered/exists in the ARIA treatment management system (TMS) (Varian Medical Systems, Inc.). If not, guidance for performing patient registration via ARIA or EPIC (Epic Systems Corp., Verona, WI) is provided in the *Help* window of the user interface (UI). Next, the user must input the appropriate medical record number (MRN) to "create" the patient in the SFW. This task interfaces with the *Capsule* in-house software, as the patient's CarePath information resides within this database and is accessible by the SFW as needed. Additionally, this supplementary software stores all necessary data to assist with workflow recovery should the SFW unexpectedly crash for any reason or should the user close the program prior to completion of the session. Finally, the user must confirm the presence of accurate patient information (name and MRN) in the upper window of the UI.

Following patient selection, the desired workflow must be selected via the "Select Wizard" tab. The *Help* window of this tab provides a useful visual aid to verify the treatment type. For the initial software release, only the "AP/PA Spine" workflow is accessible, though "AP/PA Metastasis" and "Whole Brain" workflows are currently queued for future development. The next tasks are then provided in the "Therapist Work" tab, where the user is prompted to first click the "Add Plan" button. This action triggers the automatic generation of a temporary "imaging" plan

for the patient (via Eclipse Scripting Application Programming Interface (ESAPI)), which is required to access the patient on the treatment console to acquire the cone-beam computed tomography (CBCT) image prior to creation of the real treatment plan. This imaging plan consists of an AP field delivered on a solid water phantom, containing only a single Monitor Unit (MU) as an added safety measure should the plan inadvertently be delivered with the patient on the table. The remaining tasks within this tab provide guidance for the plan approval and scheduling processes (for the imaging plan) in the appropriate workspace in ARIA, including several minor adjustments to specific plan parameters as needed.

Upon completion of the aforementioned therapist duties, the pre-patient workspace directs the user to the "Physician Work" tab, in which the on-call physician commences with treatment approval of the imaging plan. The next task involves definition of the appropriate plan and course nomenclature. For this action, the user selects the most superior and inferior vertebral body to be treated (a single vertebral body can also be chosen). Upon selection of the desired treatment area, the SFW automatically generates the appropriate clinical course and treatment plan IDs in accordance with institutional nomenclature standards. Of note, this task interfaces with code developed for the in-house *Symphony* software. Next, the user is prompted to add a prescription to the relevant clinical course, with the appropriate Course ID and prescription name available for copy/paste to ensure compliance with designated naming conventions. The platform supports both single and multi-fraction treatment courses. Conclusion of this task marks the completion of all requisite actions for the pre-patient subprocess. Importantly, all preceding steps can be executed prior to patient placement on the treatment couch (for emergent radiotherapy (RT)) or prior to patient arrival (for standard palliative RT).

S.I.B. Treatment Planning Subprocess

The treatment planning subprocess begins with the "Image Acquisition" step. Following patient setup in the treatment vault, the user must first ensure that all table values appearing on the treatment console are zeroed prior to proceeding. The patient's imaging plan can then be accessed on the LINAC console, allowing for acquisition of an extended range CBCT image of the desired treatment anatomy. After acquisition of the CBCT, the user is reminded to select the "Restore Couch" function on the treatment console (to reset the couch position following an offset due to the extended range CBCT) and to capture the table coordinates, an imperative task for ensuring proper table positioning for alignment with the intended treatment position during plan delivery. Lastly, the user must close the patient on the treatment console to enable the acquired image to be returned to the treatment planning system (TPS) (Eclipse – Varian Medical Systems, Inc.). Failure to close the patient plan on the console prompts a notification to the user prior to proceeding to the subsequent tab.

Within the "Contouring" tab, the user must first select the desired image set as verified by the date/time stamp associated with it (this will default to the most recent acquisition, but the user can select any image for the patient if multiple exist). The user then selects the "Add Structures to Contour" button, which automatically appends (via ESAPI) an external "body" contour and empty target structure within the image set in the contouring workspace of the TPS. Next, the physician is prompted to access the contouring workspace to delineate the intended treatment area. To perform this task, the physician must simply contour two slices of the CBCT image, encompassing the most superior and inferior extent of the desired treatment field. An example of such contours provided by the physician during one of the trial runs is displayed in Figure S1. The location of the superior and inferior contours sets the field length, while the left/right extent of the contours

defines the field width. Additionally, the software automatically applies block margins (from the delineated slices) of 10 mm in the left-right direction to account for the hourglass shape of parallel-opposed treatment beams (to improve the uniformity of dose coverage to the intended treatment area) and 3 mm in the superior-inferior direction to account for the beam penumbra. The physician is then instructed to save and close the contouring workspace, signifying that all tasks related to this step have been completed.

The final tasks associated with the treatment planning subprocess are performed in the "Plan Generation" tab. Dropdown menus within the main window of the UI allow the user to confirm the course and prescription (this will default to the most recent course and prescription, but the user can access any other courses or prescriptions that may exist for a given patient if they have undergone multiple courses/treatments of simulation-free RT (sim-free RT) via the SFW platform). The user is also prompted to confirm the desired plan settings, including verification of the prescription and plan parameters (structure set, target structure, and machine) and selection of the desired beam energy (6X, 10X, and 18X are available). Upon validation of the intended settings, the user selects the "Create Plan" button, and an AP/PA spine plan is automatically generated in the TPS (via direct application of ESAPI) within a matter of seconds. An example 18X plan generated by this software for a common palliative prescription of 2000 cGy in 5 fractions (400 cGy per fraction) is provided in Figure S2. For the initial "AP/PA Spine" workflow, these are rules-based 2D plans prescribed to isocenter at the patient midline. Each field is assigned a weighting of 1.000 (i.e., delivers half the prescribed dose), and the plan normalization value is set at 200.00% to achieve the intended dose. The plan's isocenter and the table coordinates captured during the CBCT acquisition are utilized to generate new table coordinates, allowing the therapists to move the patient into treatment position without necessitating manual table shift

calculations. The creation of any needed reference points along with dose tracking is also handled through ESAPI (note that all plan and reference point IDs also adhere to nomenclature standards). Additionally, the patient plan is automatically exported to a commercial surface-guidance (SG) software, enabling SGRT capabilities to further ensure proper positioning during treatment. Lastly, the physician reviews the treatment plan in the planning workspace of the TPS.

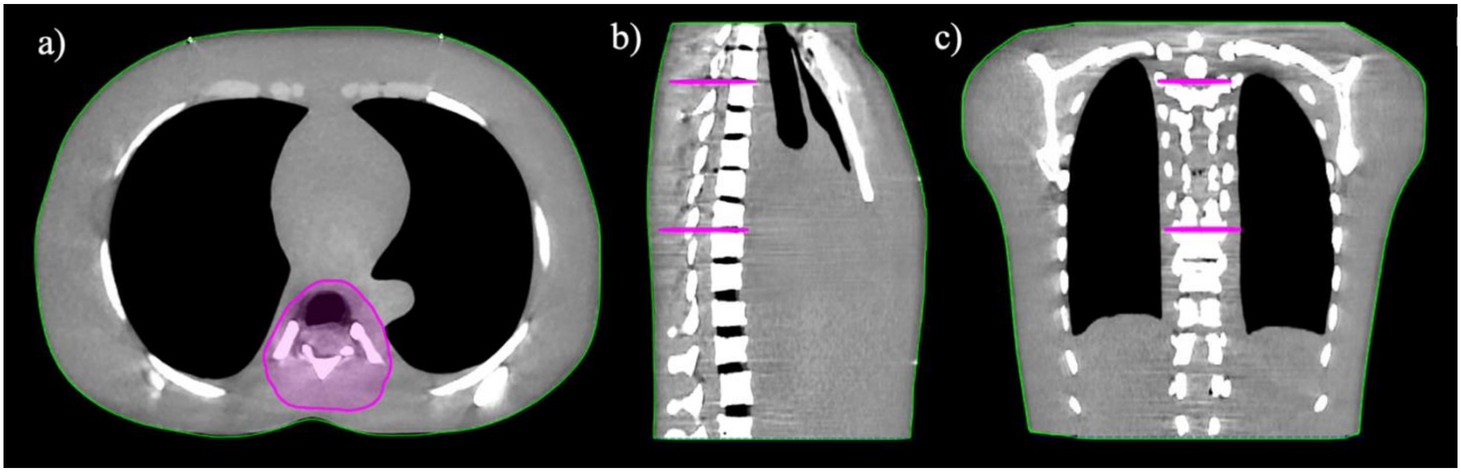

Figure S1 – Contours generated by a physician delineating the desired treatment area on the thorax phantom. Shown are a) axial, b) sagittal, and c) coronal slices, with the “target” contours displayed in pink. Note that these contours are expected to outline the most superior and inferior aspects of the treatment field, while the left-right extent of these contours determines the field width when viewed in the anterior-posterior directions.

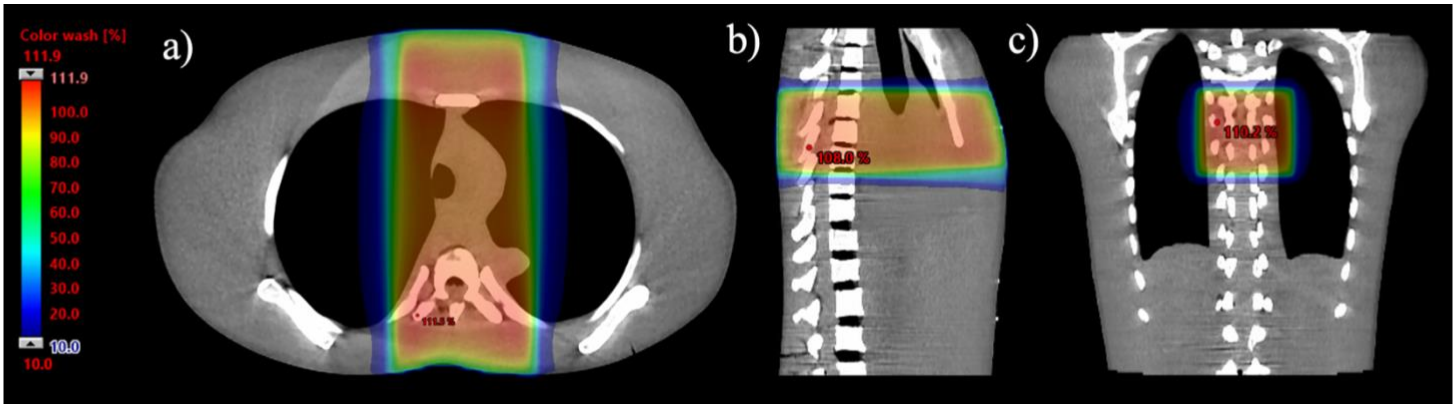

Figure S2 – Example of a simple AP/PA treatment plan generated by the in-house software on the thorax phantom. Shown are a) axial, b) sagittal, and c) coronal slices for 18X beams with a prescription of 400 cGy for 5 fractions to a total of 2000 cGy. The color-wash dose indicates regions receiving less than the maximum dose (111.9% of prescription) and greater than 10% of the prescribed dose as displayed by the slider bar on the left. The maximum dose points for each of the slices is shown in red.

S.I.C. Plan Finalization Subprocess

Plan finalization and approval following the plan generation process is initiated within the "Plan Finalization" step. Due to certain automated processes, an additional reference point may be associated with the plan that requires manual deletion by the user within the planning workspace, and the automated audit verifies that any extraneous reference points have indeed been removed. The user must then attach the treatment plan to the appropriate patient diagnosis code. Next, the user identifies the approving on-call physician from a dropdown menu and activates the "Create/Send Document" button. This action automatically generates the plan approval document with the appropriate ClearCheck (Radformation, New York, NY) template and uploads a pre-signed copy to the patient's documents workspace in ARIA using a standard File Transfer Protocol program via a vendor-provided "Web Service" API ("Gateway"). The user then completes the planning approval process as instructed. Subsequently, the user must enter the new table coordinates calculated during the plan generation process in the treatment preparation workspace of the TMS. For simplicity, the SFW directly displays these coordinates for the user. Following selection of the appropriate tolerance table for treatment, the user proceeds to schedule the plan within the TMS. To conclude the plan finalization subprocess, the physician verifies the contents of the plan approval document and executes the treatment approval task if satisfied. Note that the standard plan finalization process was reviewed during the developmental phase of the SFW and was streamlined to incorporate only the most necessary steps to reduce completion time. As the patient and any associated data are not handed off from group-to-group within the department as would normally occur, certain communication protocols inherent to a standard workflow are not required (or can be completed after the first treatment fraction via an automated journal note and simulation document).

S.I.D. Quality Assurance and Treatment Subprocess

Prior to treatment initiation, an independent MU secondary verification is performed within the "Pre-Treatment QA" tab via an in-house function incorporated into the SFW as detailed in the main manuscript text, with results displayed for the user for each unique treatment beam. The MU Second Check report can be exported as a PDF and deposited within the patient document workspace via the Varian "Gateway" as before. Additionally, an automated DICOM service is utilized to transfer the treatment plan to a commercial secondary verification software. This solution can serve as a redundancy check in instances in which the results returned by the in-house function are outside of the expected tolerance or for more complex treatments as expected in future iterations of the SFW.

Upon completion of the MU second check, an official chart check is executed. This process interfaces with another in-house platform, *Chartist*, and the checks automatically incorporate all audited checks already performed in the SFW supplemented by a manual checklist for any requisite checks that cannot be automated by the software. While the chart check contents can be viewed in the SFW, all data is auto-uploaded to the *Chartist* database as well, enabling review in that application at any time following the treatment. An example of chart check results displayed within the UI is provided in Figure S3. Following completion of the chart check, the plan is ready to deliver and the user is transferred to the "Treatment" tab.

After accessing the plan on the treatment console, the therapist is instructed to enter the treatment vault to verify (via the in-room lasers) that the patient has not moved appreciably since acquisition of the CBCT image, making adjustments as necessary. The therapist then uses the couch pendant to drive the table to the new treatment isocenter (the coordinates of which were calculated during the plan generation process as mentioned previously), and the commercial SGRT

tool is employed to verify patient positioning in room. Prior to beam-on, procedure-specific image guidance, including acquisition of planar "port films" for the proposed spine workflow, is performed to facilitate further fine-tuning of the patient setup. Following a procedural pause for the final confirmation of patient identity and relevant treatment details (site, prescription, etc.), the treatment process is initiated and the plan is delivered. The concluding task in the workflow involves the automatic generation of a journal note, documenting important procedural details for future review with varying templates determined by the number of fractions to be treated.

Results

Done	Test	Comments
☑	SimFree care path generated or selected	5/24/2024 11:32:03 AM
☑	Imaging plan created	5/24/2024 11:33:15 AM
☑	Imaging plan Treatment Approved	5/24/2024 11:36:19 AM
☑	Imaging plan scheduled in Aria	5/24/2024 11:35:38 AM
☑	Target structure added to patient	5/24/2024 11:48:42 AM
☑	Target structure has segments	5/24/2024 11:48:42 AM
☑	SimFree Treatment Plan created	5/24/2024 11:49:47 AM
☑	SimFree MU second check less than 5% difference	5/24/2024 11:56:12 AM
☑	Daily Dose correct	ONCE DAILY<br>All ref points have correct daily dose.
☑	Session Dose correct	All ref point have correct session dose.
☑	Total Dose correct	All ref point have correct total dose.
☑	Plan attached to Rx	5/24/2024 11:51:50 AM
☑	Dose Rate correct for energy/technique/machine	All beams are at max dose rate.
☑	Calculation Grid Size appropriate	5/24/2024 11:49:16 AM
☑	Rx Name correct	5/24/2024 11:38:29 AM
☑	Rx Volume names meet Nomenclature standards	5/24/2024 11:38:29 AM
☑	Plan ID meets Nomenclature standards	5/24/2024 11:49:47 AM
☑	Reference Points meet Nomenclature standards	5/24/2024 11:50:25 AM
☑	DRRs Present	All fields have a DRR.
☑	Correct # of sessions	5/24/2024 11:55:33 AM
☑	Rx Dose & Fx match plan	Plan Dose = 20 Gy<br>Rx Dose(s) = 20 Gy<br>Plan Fx = 5<br>Rx Fx = 5
☑	Rx Frequency correct	5/24/2024 11:38:29 AM
☑	Rx Energy matches plan	Plan and Rx energies match
☑	Plan approval document signed	5/24/2024 11:52:40 AM
☑	Treatment fields have time entered	5/24/2024 11:53:25 AM
☑	New table coordinates entered	5/24/2024 11:54:33 AM
☑	Proper table tolerance selected	5/24/2024 11:55:09 AM
☑	Course name inline with guidelines	5/24/2024 11:38:29 AM

Figure S3 – Results for a sample chart check conducted within the in-house platform. The displayed checks represent those that ensure that the plan has been generated as appropriate and is ready for treatment. These checks are automatically performed during the audit process following the successful completion of each task as described.

S.II. IN-HOUSE SECOND CHECK SOFTWARE VALIDATION

S.II.A. Methods

For each energy available within the platform (6X, 10X, and 18X), AP/PA treatment beams with various symmetric and asymmetric field sizes (e.g., 2 cm × 2 cm to 30 cm × 30 cm) were first delivered to simple TPS-generated virtual phantoms designed to mimic uniform solid water or that included simulated bone and lung heterogeneities (with a representative dose prescribed to midline – e.g., 800 cGy in 1 fraction). Then, to assess the performance of the in-house solution for more patient-realistic cases and to perform an initial evaluation of its potential use limitations, simple plans were created on a CBCT acquisition of the anthropomorphic thorax phantom utilized for the dry runs as previously described, with the location of the fields and calculation points placed in “lung” tissue. These plans were generated to simulate other potential palliative cases, such as the treatment of bone metastases in the ribs, occurring in the presence of lung tissue. As before, various field sizes were applied for a more comprehensive evaluation of potential use cases, though only for an energy of 6X given the treatment field in lung. Finally, to validate the secondary verification software using real CBCT patient acquisitions, plans were generated on a pediatric and several adult datasets, with various energies and field sizes utilized for fields and calculation points located in soft tissue, lung, or bone (either on the central axis or displaced laterally). All plans were then imported to the integrated second check software, and the percent difference in the MUs calculated by the TPS and this software was recorded (with an expected variation $\leq \pm$ 5.0%). Additionally, the second check results for all formally conducted dry runs were documented. A summary of the validation tests is provided in Table S1, while the data supporting this validation process are elaborated upon in the subsequent section.

Table S1 – Summary of the validation tests for the in-house monitor unit secondary verification software. Note that as the dose was prescribed to midline, the depth of the calculation point was approximately one-half the thickness of the phantom (off-axis calculation points for tests 1b – 4b were only displaced laterally).

Test	Phantom/patient	Thickness [cm]	No. plans[1]	Range of field sizes [cm]
1a	Solid water (TPS)	15.0	9	2.0 × 2.0 – 30.0 × 30.0
2a	Solid water (TPS)	40.0	9	3.0 × 3.0 – 30.0 × 30.0
3a	Solid water (TPS)	2.6	5	2.0 × 2.0 – 10.0 × 10.0
4a	Solid water (TPS)	47.0	5	3.1 × 3.4 – 10.0 × 10.0
5a	Slab (TPS)	40.0 total (7.0 lung, 5.0 bone)	5	3.1 × 8.3 – 10.0 × 10.0
6a	Thorax	~22.0	7	5.0 × 5.0 – 9.0 × 18.0
-	Thorax (dry runs)	~22.0	10	5.1 × 5.2 – 7.1 × 8.4
1b	Adult[2]	~20.0	4	5.0 × 10.0 – 12.2 × 13.4
2b	Adult[3]	~26.0	1	8.5 × 13.2 – 9.4 × 14.0
3b	Adult[4]	~22.0	6	7.0 × 11.0 – 17.0 × 29.0
4b	Pediatric	~13.0	5	2.0 × 4.0 – 11.0 × 12.0

[1] For tests 1a–5a and 1b–4b, all plans were calculated for each available energy (6X, 10X, 18X); for test 6a, plans were calculated for 6X only given the treatment fields in lung; for the dry runs, plans were calculated for only one of the available energies (6X n=2, 10X n=4, 18X n=4) based on what was selected on the platform during the trial run.

[2] Female with large breast implants
[3] Male with large separation, gas pockets present in abdominal region
[4] Male pelvic region with extended field CBCT acquisition

S.II.B. Results

The average percent deviation in MUs computed by the in-house second check software in comparison to the TPS for each of the test cases is provided in Table S2. Of note, for all validation calculations conducted on the virtual uniform solid water or heterogenous slab phantom geometries generated within the TPS (tests 1–5), all discrepancies between the TPS calculations and the secondary verification software were within ± 3.0%, with average values for each energy generally within ± 1.0%. Overall, the largest discrepancies for these phantoms were observed for cases tested at both extremes of the varied solid water thickness (2.6 cm for test 3 and 47.0 cm for test 4). Although it is anticipated that such patient separations will rarely be encountered when using this platform (particularly thicknesses < ~10 cm), these tests facilitated the validation of the software across the entire range of potential patient thicknesses in the AP/PA direction. Furthermore, the validation results on the virtual heterogenous slab phantom with simulated lung and bone tissue (test 5) demonstrated good agreement across all energies and field sizes.

For calculations performed on the anthropomorphic thorax phantom with the comparison point located in “lung” (test 6), significantly larger discrepancies were observed between the TPS and in-house software, with an average deviation of -4.50 ± 7.66 [%]. Out of the 12 total fields in which the calculated MUs were compared (2 fields for each of the 6 test plans), 6 fields exhibited deviations beyond the expected tolerance of ± 5.0%, with 4 such discrepancies exceeding 10.0%. However, given the simplistic nature of this calculation model (especially in the presence of lung heterogeneities) when compared to a more refined algorithm such as Eclipse’s AAA (or AcurosXB), such variation is anticipated for these scenarios [Fogliata et al.]. Additionally, for palliative spine RT as will be the initial application of this platform, it is unlikely that any

calculation points at midline will reside within lung tissue, and these plans were therefore included to analyze the potential limitations of this calculation algorithm for future uses.

All secondary verification results for the 10 dry runs in this study were within the expected tolerance of ± 5.0%, with 65% of fields (13/20) exhibiting deviations less than ± 1.0%. In general, the number of MUs calculated by the second check software was marginally lower than that calculated by the TPS. The magnitude of variation displayed minimal correlation with the selected energy or field sizes (despite the suggested result of higher discrepancies for 10X as seen in the final row of Table S2); however, it was observed that the location of the calculation point had a noticeable bearing on the magnitude of the calculated discrepancies.

Finally, the validation results on the clinical patient datasets exhibited variability, with the location of the calculation point seeming to have the most substantial influence on the accuracy of the computed values as before. Points calculated in lung tissue or bone tended to result in larger discrepancies. As before, no discernable trends were detected in the comparison data with respect to variation in energy or field size. For the adult patient with large breast implants (test 1b), among the 24 total beams assessed (4 plans with 2 beams each across 3 different energies), 66.7% (16/24) fell within the expected tolerance of ± 5.0%, and all values not within this tolerance occurred for calculation points in lung tissue. For the large adult patient with noticeable abdominal gas presence (test 2b), all calculation points fell in soft tissue and all 6 beams evaluated were well within ± 2.0%. When considering the male pelvic region with an extended field CBCT and overall larger field sizes (test 3b), 72.2% (26/36) of beam calculation results were within ± 5.0%. Of note, 80.0% (8/10) of values not within this range (including all values greater than ± 10.0%) occurred for calculation points in bone. Similarly, for the pediatric patient (test 4b), out of 30 total beams

evaluated, 73.3% were within tolerance, with nearly all calculations not within ± 5.0% occurring within lung tissue.

Table S2 – Validation results for the in-house monitor unit (MU) second check software. For each test as listed in Table S1, the average discrepancy in the number of calculated MUs within the secondary verification software relative to the treatment planning system (TPS) is displayed (to one standard deviation), as well as the minimum and maximum variation (in magnitude as a percentage) for any individual beam. Positive results indicate instances in which the second check software calculated a larger number of MUs than the TPS. Note that the expected tolerance is ± 5.0%.

	6X			10X			18X		
Test	Average [%]	Min	Max	Average [%]	Min	Max	Average [%]	Min	Max
1a	0.63 ± 0.23	0.41	0.97	-0.05 ± 0.29	-0.08	0.46	-0.34 ± 0.47	0.06	-1.23
2a	0.57 ± 0.57	0.00	1.32	-0.14 ± 0.38	0.08	-0.72	-0.34 ± 0.47	0.07	-0.96
3a	1.19 ± 0.23	1.01	1.53	-0.49 ± 0.51	0.22	-0.98	2.06 ± 0.81	1.49	2.63
4a	2.30 ± 1.03	0.76	2.93	1.34 ± 0.47	0.65	1.64	-0.29 ± 0.86	-0.51	0.97
5a	0.93 ± 0.36	0.77	1.36	0.01 ± 0.36	0.01	0.52	-0.47 ± 0.67	-0.13	-1.11
6a	3.25 ± 7.58	1.82	13.40	-	-	-	-	-	-
Dry run	-0.43 ± 0.81	0.40	-1.20	-2.25 ± 1.14	0.50	-3.70	0.25 ± 0.33	0.00	-3.20
1b	1.87 ± 5.02	0.00	11.18	1.42 ± 5.05	0.70	9.23	0.56 ± 4.72	0.88	7.48
2b	-0.48 ± 0.70	0.02	-0.97	-1.15 ± 0.57	-0.75	-1.55	-0.51 ± 0.61	-0.08	-0.94
3b	2.79 ± 6.50	0.45	14.27	1.37 ± 4.73	0.03	9.48	1.18 ± 3.99	0.08	-7.47
4b	2.74 ± 5.98	0.05	11.51	0.47 ± 3.38	0.00	5.69	-2.91 ± 5.11	0.19	-10.89

S.III. ADDITIONAL IMPLEMENTATION NOTES

Moving forward, staff training critical to the success of this platform's integration will continue to occur prior to and during the clinical deployment process. Throughout the initial development and training phases, many SFW platform dry runs and demonstrative sessions were passively observed by additional staff members to gain familiarity with the software prior to their own testing to reduce the rate of any associated learning curve. Comprehensive training documentation and general reference materials pertinent to this tool are accessible via a department-wide SharePoint (Microsoft Corp.) page, including a user's manual, standard procedures, workflow description, etc. Additionally, this page will provide any instructions necessary for recovering from potential operational errors encountered during this process, which may occur due to user noncompliance with platform guidance or failures within the operation of the automated processes (though this is an unexpected occurrence). For example, users will be provided with information on how to manually include the body and target contours should this automation fail for whatever reason following acquisition of the CBCT image. The initial clinical implementation of this platform will also occur for standard palliative RT cases treated during the working day only to ensure that physics is able to provide full support for the first several patient treatments utilizing this platform. Only after verifying that this process can be completed as expected with no significant issues will this tool become available for on-call, emergent procedures outside of normal working hours. Moreover, a formal prospective failure modes and effects analysis (FMEA) in accordance with guidance from the American Association of Physicists in Medicine Task Group 100 [Huq et al.] has been ongoing throughout the development of this platform. This process has facilitated the identification of potential failure modes within this workflow, enabling the incorporation of additional quality control measures to mitigate the

possibility of such failures occurring. While outside of the immediate scope of this study, the results of the supplementary FMEA will be the subject of a forthcoming manuscript to be completed upon clinical implementation of this platform.

REFERENCES

Fogliata A, Vanetti E, Albers D, et al. On the dosimetric behaviour of photon dose calculation algorithms in the presence of simple geometric heterogeneities: comparison with Monte Carlo calculations. *Phys Med Biol*. 2007;52(5):1363-1385. doi:10.1088/0031-9155/52/5/011

Huq MS, Fraass BA, Dunscombe PB, et al. The report of Task Group 100 of the AAPM: Application of risk analysis methods to radiation therapy quality management. *Med Phys*. 2016;43(7):4209. doi:10.1118/1.4947547
